# Intracranial approach for sub-second monitoring of neurotransmitters during DBS electrode implantation does not increase infection rate

**DOI:** 10.1371/journal.pone.0271348

**Published:** 2022-08-22

**Authors:** Brittany Liebenow, Michelle Williams, Thomas Wilson, Ihtsham ul Haq, Mustafa S. Siddiqui, Adrian W. Laxton, Stephen B. Tatter, Kenneth T. Kishida

**Affiliations:** 1 Neuroscience Graduate Program, Wake Forest School of Medicine, Winston-Salem, NC, United States of America; 2 Department of Physiology and Pharmacology, Wake Forest School of Medicine, Winston-Salem, NC, United States of America; 3 Department of Neurosurgery, Wake Forest School of Medicine, Winston-Salem, NC, United States of America; 4 Department of Neurology, University of Miami Miller School of Medicine, Miami, FL, United States of America; 5 Department of Neurology, Wake Forest School of Medicine, Winston-Salem, NC, United States of America; Murdoch University, AUSTRALIA

## Abstract

**Introduction:**

Currently, sub-second monitoring of neurotransmitter release in humans can only be performed during standard of care invasive procedures like DBS electrode implantation. The procedure requires acute insertion of a research probe and additional time in surgery, which may increase infection risk. We sought to determine the impact of our research procedure, particularly the extended time in surgery, on infection risk.

**Methods:**

We screened 602 patients who had one or more procedure codes documented for DBS electrode implantation, generator placement, programming, or revision for any reason performed at Wake Forest Baptist Medical Center between January 2011 through October 2020 using International Classification of Diseases (ICD) codes for infection. During this period, 116 patients included an IRB approved 30-minute research protocol, during the Phase 1 DBS electrode implantation surgery, to monitor sub-second neurotransmitter release. We used Fisher’s Exact test (FET) to determine if there was a significant change in the infection rate following DBS electrode implantation procedures that included, versus those that did not include, the neurotransmitter monitoring research protocol.

**Results:**

Within 30-days following DBS electrode implantation, infection was observed in 1 (0.21%) out of 486 patients that did not participate in the research procedure and 2 (1.72%) of the 116 patients that did participate in the research procedure. Notably, all types of infection observed were typical of those expected for DBS electrode implantation.

**Conclusion:**

Infection rates are not statistically different across research and non-research groups within 30-days following the research procedure (1.72% vs. 0.21%; p = 0.0966, FET). Our results demonstrate that the research procedures used for sub-second monitoring of neurotransmitter release in humans can be performed without increasing the rate of infection.

## Introduction

There is great promise in leveraging opportunities in the operating room (OR) to conduct human neuroscience research. Deep Brain Stimulation (DBS) in particular lends itself to research, as the procedure typically entails intraoperative electrophysiological assessments of neural targets; thus, research data can be acquired with relatively minor protocol changes [1–5]. This has allowed research teams to make breakthrough discoveries using data collected in the OR during DBS electrode implantation procedures [1–16]. Notable innovations include first-of their kind measurements of neurotransmitters [1–5,7,8], including dopamine [1,2,4,5,12], serotonin [3,4,12], and adenosine [7,8] in humans. Other advances include single unit recordings from substantia nigra [6] and expanding DBS targeting to provide symptom relief in treatment-refractory depression, substance use disorder, Alzheimer’s disease, and obsessive-compulsive disorder [9–11,13–16].

One potential limiting factor in conducting translational research in the OR is the possibility that the added OR time necessary to conduct experiments may increase infection risk [17,18]. Infection risks following DBS surgeries are well described and provide a good basis for comparison. A metanalysis covering 1354 patients across 23 articles reported a 6.9% overall risk of infection following DBS electrode implantation surgeries [19]. Similarly, one large single-center study of 447 DBS patients identified an overall infection rate of 5.82% (26 patients); this study also identified a 30-day infection rate of 2.01% (9 patients) [17]. This 30-day infection rate is corroborated by other large, single-institution studies, including a study of 273 patients with a median time to infection of 1 month that reported an infection rate of 3.1% across procedures for primary DBS electrode placement [20]. To date, however, no study has investigated whether adding OR time due to a predefined research protocol increases infection risk after elective surgery.

Our group has over a decade of data and experience measuring neurotransmitters while patients complete behavior tasks during DBS electrode implantation surgeries [1–5,12]. These experiments have added a maximum of 30 minutes to the scheduled OR time. Thus, we have ample research and surgical records to retrospectively explore whether there are group differences in post-operative infection rates between patients receiving DBS who participate in research (‘research’) and who do not participate in research (‘non-research). Here, we compare the 30-day post-operative infection rates of research (N = 116) and non-research (N = 486) groups at our institution to investigate whether these experiments increased infection risk.

## Materials and methods

The Institutional Review Board at Wake Forest University Health Sciences approved all procedures described for this retrospective study (IRB00064371) and for our ongoing research protocol in the DBS operating room (IRB00017138). Data were analyzed anonymous under IRB00064371. Research participants gave their written informed consent prior to participation in the research study under protocol IRB00017138. All relevant data for sharing are contained within the manuscript and its Supporting Information files.

### Clinical data

We screened all patients who had one or more procedure codes documented for DBS electrode implantation, generator placement, programming, or revision for any reason at Wake Forest Baptist Medical Center between January 2011 through October 2020 using International Classification of Diseases (ICD) codes for infection. These ICD codes included: T85.731 (Infection and inflammatory reaction due to implanted electronic neurostimulator of brain); T85.734 (Infection and inflammatory reaction due to implanted electronic neurostimulator, generator), 61867 (First Electrode with microelectrode recording, typical), 61868 (Second Electrode on same side with recording, other side), 95983 (Intraoperative analysis / programming), 61885 (For single electrode), 61886 (For multiple electrodes), 61880 (Electrode removal / revision), 61888 (Generator removal / revision, use for attaching previously placed lead); Z45.42 (DBS Phase / Stage 3, generator change).

We further screened the total number of DBS procedures using the medical record number (MRN) of all patients who received DBS and participated in our research protocol (Table 1).

**Table 1:** Yearly patients consented for DBS research surgeries.

Year	Total Patients Consented	Total Patients Completed	Total Patients Consented Only	% Patients Completed	% Patients Consented Only
*2012*	4	4	0	*100%*	*0%*
*2013*	23	17	6	*74%*	*26%*
*2014*	20	14	6	*70%*	*30%*
*2015*	9	7	2	*78%*	*22%*
*2016*	13	11	2	*85%*	*15%*
*2017*	14	10	4	*71%*	*29%*
*2018*	24	18	6	*75%*	*25%*
*2019*	23	15	8	*65%*	*35%*
*2020*	9	8	1	*89%*	*11%*

Total numbers of patients who are consented for, and ultimately complete, a research protocol during deep brain stimulation (DBS) neurosurgery. This data starts in April 2012 and continues through December 2020.

Our research procedure occurs during microelectrode-based electrode placement planning for deep brain stimulation (DBS) surgery, which will be summarized here [1–4,12]. In preparation for deep brain stimulation (DBS) surgery, the neurosurgeons on our research team fit a a Cosman–Roberts–Wells (CRW) stereotactic frame on the head, and the medical team takes a volumetric computed tomography (CT) scan. This information is integrated with preoperative MRI images, which are used together to determine DBS electrode targets. Therapeutic targets are the subthalamic nucleus, internal segment of the globus pallidus, or the thalamus. Our research targets (the caudate, putamen, and thalamus) are anatomically superior to therapeutic targets used in DBS, which are approximately 15mm to 20mm deeper than the research recording targets [1–4,12]. Thus, use of our research electrode is typically performed just prior to functional mapping with tungsten microelectrodes (provided by FHC inc.) [1–4,12].

The neurosurgeons place our custom carbon-fiber microsensors into the caudate or putamen, using one of five potential microelectrode recording trajectories made available by a five-hole “Ben-gun” array, at depths that do not surpass treatment depths deemed safe during planning stages. The carbon-fiber microsensors are constructed by our laboratory to match the dimensions of the tungsten microelectrodes used for functional mapping during DBS electrode implantation surgery. These custom carbon fiber microelectrodes have passed a successful Ethylene Oxide Sterilization Exposure and Sterility Audit conducted by BioLabs to ensure preoperative ethylene oxide treatment fully sterilizes the carbon-fiber microsensor electrodes. These electrodes have also been validated and approved for autoclave and hydrogen peroxide sterilization.

The research protocol adds a maximum of 30 additional minutes to the DBS surgery time and requires an informed consent process that takes place ahead of the surgery [1–4,12]. This compares to an average overall DBS operating time previously reported as 4 hours for unilateral and 4.7 hours for bilateral surgeries [21]. While the research team measures neurotransmitter levels, participants complete behavioral tasks displayed on a computer monitor and input decisions using a standard gaming controller [1–4,12]. Research records were also screened to determine the number of patients consenting to versus completing research protocols during their DBS surgeries.

### Statistics

All statistical analyses were conducting using RStudio [22]. We used Fisher’s Exact test (FET) to compare 30-day infection rates following Phase 1 DBS electrode implantation procedures across patients that did and did not participate in our DBS research protocol during their Phase 1 DBS procedure.

## Results

**Fig 1:**
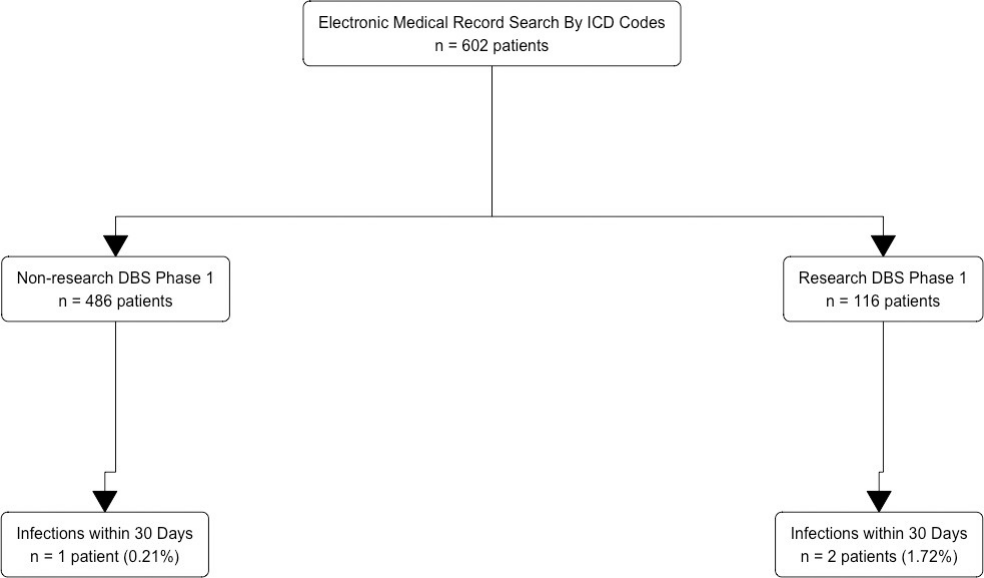
Acquisition of cases by medical record review. Flowchart demonstrating the acquisition and filtering of deep brain stimulation (DBS) procedures (or cases) from the electronic medical record (EMR) using International Classification of Diseases (ICD) codes. There is no statistically significant difference between the non-research and research DBS groups in the 30-day infection rates (0.21% vs. 1.72%; p = 0.0966, FET).

We identified 602 patients who had a Phase 1 DBS electrode implantation procedure performed at Wake Forest Baptist Medical Center between January 2011 through October 2020 using our specific ICD codes for infection (Fig 1). Of those 602 patients, 486 patients met criteria for having a Phase 1 DBS procedure that did not include the research protocol (‘non-research’), and an additional 116 patients met criteria for Phase 1 DBS procedures that did include the research protocol (‘research’).

Of the 486 non-research DBS patients, there was 1 infection within 30 days of the DBS procedure (0.21% of patients). Of the 116 research DBS patients, there were 2 within 30 days of the DBS procedure (1.72% of patients, Fig 1). Using FET, we determined that there is no statistically significant difference between the non-research and research DBS groups in the 30-day infection rates (0.21% vs. 1.72%; p = 0.0966, FET, Fig 1).

The infectious pathogens in the research group were reported to be: Methicillin-resistant Staphylococcus aureus (MRSA), Methicillin-sensitive Staphylococcus aureus (MSSA), and Serratia marcescens. All of these pathogens have been reported in the literature as potential causes of post-operative infections after DBS, with the Staphylococcus genus the most common culprit [23].

We also identified the yearly number of patients who were approached for consent to participate in research, and the actual number of patients who completed the research study starting in April 2012 and continuing through December 2020 (Table 1). The diagnoses of consented patients were also identified (Table 2). Reasons for consenting but not completing a research protocol can be influenced by the individual anatomy of each participant (i.e. no good trajectory connecting striatum and target).

**Table 2:** Diagnoses of patients consented for DBS research surgeries.

Diagnosis	Total Patients Consented	Total Patients Completed	Total Patients Consented Only	% Patients Completed	% Patients Consented Only
*Parkinson’s Disease*	97	73	24	*75%*	*25%*
*Essential Tremor*	36	28	8	*78%*	*22%*
*Dystonia*	6	3	3	*50%*	*50%*

Diagnoses of patients who are consented for, and ultimately complete, a research protocol during deep brain stimulation (DBS) neurosurgery. This data starts in April 2012 and continues through December 2020.

## Discussion/Conclusion

Our results investigating the infection rates of DBS surgery patients with and without a 30-minute invasive research procedure during DBS electrode implantation demonstrate that there is no difference in 30-day infection rates between groups. This is consistent with the conclusion that these experiments can be performed without increasing the risk of infection in these patient populations. Further confidence in our results can be found in a comparison to studies reporting similar 30-day infection rates to our results. Our 30-day infection rates of 0.21% for non-research and 1.72% for research procedures are comparable to—and even lower than—similar studies reporting 30-day infection rates of 2.01% [17] and 3.1% [20] where intracranial research was not performed. The infectious pathogens reported for the research infections (MRSA, MSSA, and Serratia marcescens) have all been reported as potential infectious causes in post-operative DBS infections [23]. This provides further support that the 30-minute research period introduces no new infection related risks to patients.

There are a number of potentially influential factors in maintaining low infection rates while conducting translational research in the DBS OR. First and foremost, all research electrodes in our study go through independently validated sterilization procedures typical of all surgical equipment requiring sterilization [1–4]. During the research procedure, the neurosurgeon leads the clinical staff and maintains the sterile field while handling all equipment within the sterile field. We have limited the research protocol to a maximum of thirty minutes of additional time in surgery. Should the research tasks be delayed or extended for any reason the research activities are to be prematurely terminated at the thirty-minute threshold. This is done primarily to avoid unbounded delays in the surgery so that patient safety and comfort are maintained as much as possible. All patients who are candidates for DBS-electrode implantation surgery are offered the opportunity to participate. Those that choose to volunteer may be among the most likely to be capable of post-surgical selfcare that would aide in minimizing post-surgical infections. In this retrospective analysis, we do not have the appropriate data to assess this possibility, but it is potentially a major factor in the low infection rates we observe. The similarly low infection rates in the non-research group suggests that if this were the explanation for low infection rates then the comprehensive process of screening potential candidates for DBS-surgery at our institution would be the causative factor.

Research, including this current study, that shares information about the risks of observational human research studies during DBS are necessary to verify the anticipated safety of new applications of intracranial research protocols in the neurosurgery setting. The DBS-electrode implantation procedure is a safe and relatively low risk procedure as has been demonstrated repeatedly in the past [1–5,12]; it also affords unique access to areas of the human brain that have not been accessible in the past. Measurements of neurotransmitters, single units, and local field potentials in the human brain may provide new information about brain function and the mechanisms underlying disorders that may aid in improving the efficacy of DBS treatment [1–8] or in the development of novel neurosurgical goals. In addition to basic knowledge generation, these studies may also lead to the development of novel biologic markers of disease and treatment management. Our demonstration that these kinds of research protocols can be performed without an increase in infection rates should–with appropriate expertise, care, and consideration–encourage further intracranial investigation of human brain function. Our results show that intracranial recordings of sub-second neurotransmitter release in a time-extending research protocol utilizing a novel research probe are possible without increasing infection rates.

## Supporting information

S1 File Anonymized patient list. This file includes a list of all patients included in our study. There are separate columns for infection and whether a patient was a study patient, indicated with a “1”. The days to infection are included with a countdown to the prior surgery and a countdown to the study (if applicable). The culture information is also included in the final column for patients with infections.
(XLSX)

S2 File Anonymized patient list of infected patients only. This file includes only the patients with infections included in our study. There are separate columns for type of surgery before infection and study participation (indicated with a “1”). The days to infection are included with a countdown to the prior surgery, a countdown to the prior phase 1 surgery, a countdown to the study (if applicable). The culture information is also included in the final column.
(XLSX)

## References

[1] Kishida KT, Sandberg SG, Lohrenz T, Comair YG, Sáez I, Phillips PEM, et al. Sub-second dopamine detection in human striatum. PLoS One [Internet]. 2011;6(8):e23291. Available from: http://www.ncbi.nlm.nih.gov/sites/entrez?Db=pubmed&DbFrom=pubmed&Cmd=Link&LinkName=pubmed_pubmed&LinkReadableName=RelatedArticles&IdsFromResult=21829726&ordinalpos=3&itool=EntrezSystem2.PEntrez.Pubmed.Pubmed_ResultsPanel.Pubmed_RVDocSum. doi: 10.1371/journal.pone.0023291 21829726 PMC3150430

[2] Kishida KT, Saez I, Lohrenz T, Witcher MR, Laxton AW, Tatter SB, et al. Subsecond dopamine fluctuations in human striatum encode superposed error signals about actual and counterfactual reward. Proc Natl Acad Sci U S A [Internet]. 2016;113(1):200–5. Available from: http://www.ncbi.nlm.nih.gov/sites/entrez?Db=pubmed&DbFrom=pubmed&Cmd=Link&LinkName=pubmed_pubmed&LinkReadableName=RelatedArticles&IdsFromResult=26598677&ordinalpos=3&itool=EntrezSystem2.PEntrez.Pubmed.Pubmed_ResultsPanel.Pubmed_RVDocSum. doi: 10.1073/pnas.1513619112 26598677 PMC4711839

[3] Moran RJ, Kishida KT, Lohrenz T, Saez I, Laxton AW, Witcher MR, et al. The Protective Action Encoding of Serotonin Transients in the Human Brain. Neuropsychopharmacology. 2018. doi: 10.1038/npp.2017.304 29297512 PMC5916372

[4] Bang D, Kishida KT, Lohrenz T, White JP, Laxton AW, Tatter SB, et al. Sub-second Dopamine and Serotonin Signaling in Human Striatum during Perceptual Decision-Making. Neuron. 2020. doi: 10.1016/j.neuron.2020.09.015 33049201 PMC7736619

[5] Platt ML, Pearson JM. Dopamine: Context and counterfactuals. Proceedings of the National Academy of Sciences of the United States of America. 2016. doi: 10.1073/pnas.1522315113 26699497 PMC4711875

[6] Zaghloul KA, Blanco JA, Weidemann CT, McGill K, Jaggi JL, Baltuch GH, et al. Human substantia nigra neurons encode unexpected financial rewards. Science (80-). 2009. doi: 10.1126/science.1167342 19286561 PMC2839450

[7] Chang SY, Kim I, Marsh MP, Jang DP, Hwang SC, Van Gompel JJ, et al. Wireless fast-scan cyclic voltammetry to monitor adenosine in patients with essential tremor during deep brain stimulation. Mayo Clinic Proceedings. 2012. doi: 10.1016/j.mayocp.2012.05.006 22809886 PMC3538486

[8] Bennet KE, Tomshine JR, Min HK, Manciu FS, Marsh MP, Paek SB, et al. A diamond-based electrode for detection of neurochemicals in the human brain. Front Hum Neurosci. 2016. doi: 10.3389/fnhum.2016.00102 27014033 PMC4791376

[9] Bari AA, Mikell CB, Abosch A, Ben-Haim S, Buchanan RJ, Burton AW, et al. Charting the road forward in psychiatric neurosurgery: proceedings of the 2016 American Society for Stereotactic and Functional Neurosurgery workshop on neuromodulation for psychiatric disorders. J Neurol Neurosurg Psychiatry [Internet]. 2018 Aug 1;89(8):886 LP– 896. Available from: http://jnnp.bmj.com/content/89/8/886.abstract. 10.1136/jnnp-2017-317082 PMC7340367 29371415

[10] Mian MK, Campos M, Sheth SA, Eskandar EN. Deep brain stimulation for obsessive-compulsive disorder: Past, present, and future. Neurosurg Focus. 2010. 10.3171/2010.4.FOCUS10107 20672912

[11] Bourne SK, Eckhardt CA, Sheth SA, Eskandar EN. Mechanisms of deep brain stimulation for obsessive compulsive disorder: Effects upon cells and circuits. Frontiers in Integrative Neuroscience. 2012. doi: 10.3389/fnint.2012.00029 22712007 PMC3375018

[12] Read Montague P, Kishida KT. Computational underpinnings of neuromodulation in humans. Cold Spring Harb Symp Quant Biol. 2018. doi: 10.1101/sqb.2018.83.038166 31023828 PMC6736750

[13] Laxton AW, Tang-Wai DF, McAndrews MP, Zumsteg D, Wennberg R, Keren R, et al. A phase i trial of deep brain stimulation of memory circuits in Alzheimer’s disease. Ann Neurol. 2010. doi: 10.1002/ana.22089 20687206

[14] Lozano AM, Fosdick L, Chakravarty MM, Leoutsakos JM, Munro C, Oh E, et al. A Phase II Study of Fornix Deep Brain Stimulation in Mild Alzheimer’s Disease. J Alzheimer’s Dis. 2016. doi: 10.3233/JAD-160017 27567810 PMC5026133

[15] Lozano AM, Mayberg HS, Giacobbe P, Hamani C, Craddock RC, Kennedy SH. Subcallosal Cingulate Gyrus Deep Brain Stimulation for Treatment-Resistant Depression. Biol Psychiatry. 2008. doi: 10.1016/j.biopsych.2008.05.034 18639234

[16] Kennedy SH, Giacobbe P, Rizvi SJ, Placenza FM, Yasunori N, Mayberg HS, et al. Deep brain stimulation for treatment-resistant depression: Follow-up after 3 to 6 years. Am J Psychiatry. 2011. doi: 10.1176/appi.ajp.2010.10081187 21285143

[17] Tolleson C, Stroh J, Ehrenfeld J, Neimat J, Konrad P, Phibbs F. The factors involved in deep brain stimulation infection: A large case series. Stereotact Funct Neurosurg. 2014. 10.1159/000362934 25096381

[18] Valentini LG, Casali C, Chatenoud L, Chiaffarino F, Uberti-Foppa C, Broggi G. Surgical site infections after elective neurosurgery: A survey of 1747 patients. Neurosurgery. 2008. doi: 10.1227/01.NEU.0000311065.95496.C5 18300895

[19] Kashanian A, Rohatgi P, Chivukula S, Sheth SA, Pouratian N. Deep Brain Electrode Externalization and Risk of Infection: A Systematic Review and Meta-Analysis. Operative Neurosurgery. 2021. doi: 10.1093/ons/opaa268 32895713 PMC8324247

[20] Pepper J, Zrinzo L, Mirza B, Foltynie T, Limousin P, Hariz M. The Risk of Hardware Infection in Deep Brain Stimulation Surgery Is Greater at Impulse Generator Replacement than at the Primary Procedure. Stereotact Funct Neurosurg. 2013. 10.1159/000343202 23207787

[21] Blomstedt P, Hariz MI. Hardware-related complications of deep brain stimulation: A ten year experience. Acta Neurochir (Wien). 2005. doi: 10.1007/s00701-005-0576-5 16041470

[22] R Studio Team. R Studio. R.S. ed. http://www.rstudio.com/. 2020.

[23] Bhatia S, Zhang K, Oh M, Angle C, Whiting D. Infections and hardware salvage after deep brain stimulation surgery: A single-center study and review of the literature. Stereotact Funct Neurosurg. 2010. doi: 10.1159/000303528 20357522

